# Extrachromosomal circular DNAs in the differentiation of human bone marrow mesenchymal stem cells

**DOI:** 10.1186/s13287-025-04516-x

**Published:** 2025-07-18

**Authors:** Yuxi Gu, Yidan Song, Shuhua Wang, Jun Liu

**Affiliations:** State Key Laboratory of Oral Diseases & National Center for Stomatology & National Clinical Research Center for Oral Diseases, Department of Orthodontics, West China Hospital of Stomatology, Sichuan University, Chengdu, 610041 Sichuan People’s Republic of China

## Abstract

**Background:**

Extrachromosomal circular DNA (eccDNA) plays a significant role in cancer development. However, our understanding of its role in normal cells is limited. This study aimed to explore the roles of eccDNA in the differentiation of human bone marrow mesenchymal stem cells (BMSCs).

**Methods:**

Through circular DNA sequencing (Circle-seq) and computational analysis, we documented eccDNAs in human BMSCs and their differentiation into osteoblasts, adipocytes, and chondrocytes. Next, the assay for transposase-accessible chromatin with high throughput sequencing (ATAC-seq) and RNA sequencing (RNA-seq) data were integrated with Circle-seq data. The roles of phosphatidylinositol 4-kinase alpha (PI4KA) and eccDNA as functional enhancers in human BMSC differentiation were assessed in vitro.

**Results:**

Our results demonstrate that eccDNA is common in human BMSCs. In the differentiated groups including osteoblasts, adipocytes and chondrocytes, eccDNA-encoded genes presented higher expression compared to the background. We found eccDNA displayed enhanced chromatin accessibility in human BMSCs, which contribute to increased transcription of genes, such as runt-related transcription factor 2 (RUNX2), a key transcription factor for BMSC osteogenesis. We further found PI4KA, an eccDNA-encoded gene, promoted osteogenic differentiation of human BMSCs via the PI3K/AKT/mTOR pathway. Finally, we demonstrated eccDNA as functional enhancers to regulate BMSC differentiation in a dose-dependent manner.

**Conclusions:**

Our study provides the evidence of eccDNA regulating BMSC differentiation and highlights the roles of eccDNA as transcription template and enhancer in normal cells, which will facilitate future research and clinical applications.

**Supplementary Information:**

The online version contains supplementary material available at 10.1186/s13287-025-04516-x.

## Introduction

Bone marrow mesenchymal stem cells (BMSCs) are multipotent cells capable of differentiating into osteoblasts (OBs), adipocytes (ACs) and chondrocytes (CCs). Several key regulators for MSC differentiation have been reported, such as runt-related transcription factor 2 (RUNX2) and peroxisome proliferator-activated receptor gamma (PPARG) [1, 2]. Besides, researches on epigenomic regulation and genome conformation highlight the distinct genetic regulatory characteristics in MSC differentiation [3, 4].

Extrachromosomal circular DNAs (eccDNAs), independent of chromosomes, are double-stranded circular DNA and have been discovered in different species including humans [5–7]. EccDNAs range from approximately 100 bases to several megabases in size, mapping to the entire genome [5, 7, 8], and exist in both human normal and cancer cell lines [6, 9, 10]. EccDNAs can be categorized into different types, such as extrachromosomal DNA (ecDNA), small polydispersed circular DNA (spcDNA), microDNA and telomeric circles (t-circles) [7]. All of these types are referred as eccDNA in this study. It has been found that eccDNAs are prevalent in cancer genomes and play a crucial role in cancer development [6, 11, 12]. EccDNAs promote accessible chromatin and oncogene expression [6]. A cluster of approximately 10–100 eccDNAs facilitates intermolecular enhancer-promoter interactions, leading to amplified oncogene expression [12]. However, the function of eccDNAs in healthy individuals and normal physiological processes remains unclear, although they may be associated with aging [13, 14] and immune responses [9].

Previous study has reported eccDNAs in human adipose stem cells [15]. The eccDNA pattern in the adipose stem cells changes during aging and eccDNA may be potential biomarkers for stem cell aging. Several eccDNAs have been found in hBMSCs and may play a significant role in pathways connected to age-related osteoporosis progression [16]. In chondrocytes from osteoarthritis, eccDNAs may be involved in the regulation of cell degeneration [17]. However, the landscape and function of eccDNAs in stem cell differentiation are still unknown.

Phosphatidylinositol 4-kinases (PI4Ks) are enzymes that produce Phosphatidylinositol 4-phosphate (PI4P), which is a precursor of phosphatidylinositol 4,5-bisphosphate (PI(4,5)P2) in the plasma membrane [18]. PI(4,5)P2 is essential for cells to as regulatory lipids to regulate ion channels and transporters [19]. PI4KA is the main source of PI4P and maintains the PI(4,5)P2 pool in plasma membrane, which plays an essential and complex role in cells [18–20]. However, the role of PI4KA in osteogenesis of BMSCs is unknown.

In this study, we document eccDNAs in the differentiation of human BMSCs by circular DNA sequencing (Circle-seq). By combing Circle-seq data with assay for transposase-accessible chromatin with high throughput sequencing (ATAC-seq), RNA sequencing (RNA-seq) and enhancer data, we reveal the mechanisms by which eccDNA contributes to BMSC differentiation, and discover the PI4KA/PI3K/AKT/mTOR pathway to regulate BMSC osteogenesis. Our study provides new insights into the molecular mechanisms of stem cell differentiation and fate determination.

## Methods

### Cell culture and differentiation induction

Human BMSCs (HUM-iCell-s011, iCell Bioscience Inc., Shanghai) were cultured using serum-free culture system of primary mesenchymal stem cells (PriMed-iCell-012-SF, iCell Bioscience Inc.). The cells were induced to differentiation at 80% confluence. Differentiation of BMSCs were induced by iCell mesenchymal stem cell osteogenic differentiation medium (iCell-MSCYD-002, iCell Bioscience Inc.), iCell mesenchymal stem cell adipogenic differentiation medium (iCell-MSCYD-004, iCell Bioscience Inc.) and iCell mesenchymal stem cell chondrogenic differentiation medium (iCell-MSCYD-003, iCell Bioscience Inc.), respectively, for 14 days. Alizarin red s staining, alkaline phosphatase staining, oil red o staining, alcian blue staining and quantitative real-time polymerase chain reaction (qRT-PCR) of corelated marker genes were performed after induction. Insulin like growth factor 1 (IGF-1) and phosphoinositide 3-kinase (PI3K) / protein kinase B (AKT) / mechanistic target of rapamycin kinase (mTOR) -IN-2 were purchased from MedChemExpress (HY-P7018 and HY-146751).

### RNA isolation and RT-qPCR

Total RNA of cells was isolated using TRIzol Reagent (Invitrogen Corporation, CA, USA). The NanoDrop ND-1000 spectrophotometer (NanoDrop Technologies, USA) was used to detect RNA concentration and quality. ReverTra Ace® qPCR RT Master Mix (Toyobo) was used to obtain cDNA. The RT-qPCR was conducted with SYBR® green realtime PCR Master Mix (Toyobo). Relative quantification was normalized and calculated by GAPDH using 2^−ΔΔCt^. For the comparison of eccDNA-encoded gene expression in CC and undifferentiated groups, the seven genes were selected randomly by R version 4.2.3 from all entire genes on eccDNA^chr6:10523673^^–18078112^. The primer sequences used in this study are listed in the Supplementary Materials.

### Circle-Seq, ATAC-seq and RNA-seq library preparation, sequencing and analysis

Circle-Seq was conducted on uBMSCs (n = 3), OBs (n = 3), CCs (n = 3) and ACs (n = 3) by CloudSeq Biotech Inc. (Shanghai, China). The cells in each sample were mixed with proteinase K (ThermoFisher, E00491) in L1 solution (A&A Biotechnology, 010–50) and incubated at 50 °C for 16 h with agitation. Then samples were disposed following the published procedures^7^ using column chromatography on an ion exchange membrane column (Plasmid Mini AX; A&A Biotechnology). FastDigest MssI (Thermo Scientifific, ER1341) and Plasmid-Safe ATP-dependent DNase (E3110K, Plasmid-Safe ATP-dependent DNase, Lucigen) was used to purify DNA from uBMSCs, OBs, CCs and ACs. We used GenSeq® RCA DNA Amplification Kitfor phi29 amplification (Genseq Inc., GS-MB-002S) and MinElute Reaction Cleanup Kit (Qiagen) for purification. GenSeq® Rapid DNA Lib Prep Kit (GenSeq Inc.) was used for library preparation and Illumina NovaSeq 6000 with 150 bp paired end mode was used for sequencing. Q30 was used to perform quality control after harvesting paired-end reads. Low quality reads were removed by Cutadapt software (v1.9.1) and reference genome (Feb. 2009 GRCh37 / hg19) alignment of the clean reads of the high quality was conducted by bwa software v(v0.7.12). Circle-map software (v1.1.4) was used for eccDNA detectation and samtools (v0.2) was used to obtain the raw soft-clipped read counts of break point. Bedtools (v2.27.1) was used for eccDNA annotation.

For ATAC-seq, cell lysis buffer was used to treat approximately 5 × 10^4^ cells, followed by centrifugation for 10 min at 500 g. The nuclei was isolated after discarding the cytoplasmic supernatant. The nuclei were then subjected to transposition using the transposition mix (Vazyme, Hyperactive ATAC-Seq Library Prep Kit) at 37 °C for 30 min with agitation at 1,000 rpm. DNA Extraction Beads (Vazyme, Hyperactive ATAC-Seq Library Prep Kit) was used to recover the tagmented DNA and the tagmented DNA was subsequently amplified via PCR. After enrichment and quantification, the library products were sequenced on the DNBSEQ-T7 (MGI) platform.

Total RNA of cells was obtained by TRIzol Reagent (Invitrogen). NanoDrop ND-1000 (NanoDrop) was used to quantify the RNA amount and purity. Bioanalyzer 2100 (Agilent, USA) and electrophoresis was used to assess the RNA integrity. Dynabeads Oligo (dT)25–61005 (Thermo Fisher, USA) was used to obtain Poly (A) RNA. SuperScript™ II Reverse Transcriptase (Invitrogen, USA) was used to create the cDNA. The heat-labile UDG enzyme (NEB, cat.m0280, USA) was used and the PCR condition: 95℃ for 3 min; 8 cycles of 98℃ for 15 s, 60℃ for 15 s, 72℃ for 30 s; and 72℃ for 5 min. The illumina Novaseq™ 6000 (LC-Bio Technology CO., Ltd., Hangzhou) was used for the 2 × 150 bp paired-end sequencing (PE150). Quality control was conducted by fastp software. Read were mapped by HISAT2. R package edgeR was used for the identification of differentially expressed genes.

### Transmission electron microscope (TEM)

The cell precipitation was collected and the electron microscope fixative (Leagene, DF0156) was added for 2-4 h. After centrifuging, the supernatant was discarded and 0.1 M phosphate buffer PB (PH7.4) was added for 3 washes, 3 min each time. The 1% agarose solution was added into the EP tube and the sample was suspended and wrapped in the agarose. Then 0. 1% osmic acid prepared with 1 M phosphate buffer PB (PH7.4) was added for 2 h. Next, the sample was dehydrated in ascending (30%, 50%, 70%, 80%, 95%vand 100%) ethanol series for 20 min each and 100% acetone twice for 15 min each time. Next, the sample was placed in Embed 812 resin at 60 °C oven for 48 h. Leica UC7 ultramicrotome was used for section at 60–80 nm. The sections were then picked up on a copper grid and post-stained without light in 2% uranium acetate for 8 min and 2.6% lead citrate for 8 min. Images were captured using a HITACHI HT7800/HT7700TEM.

### Data collection

Transcript information was from Esembl Genes in table browser [21]. RNA expression data of ACs and CCs was collected from GSE135775 and GSE109503 [22–24]. ATAC-seq data of OBs, ACs and CCs was obtained from GSE151324 and GSE214394 [3, 25, 26]. To compare ATAC-seq signal between eccDNA and chrDNA, the ATAC-seq signal was normalized by segment length and numbers. Enhancers were from GeneHancer and EnhancerAtlas [27, 28].

### Identification of eccDNA-related genes

For eccDNA-encoded gene identification, bedtools were applied to identify full transcripts on eccDNAs. For eccDNA-regulated gene identification, bedtools were applied to identify full enhancer elements on eccDNAs. The gene targets of each enhancer were ranked by the score for gene-enhancer interaction (S_GE_) [27] and the gene with the highest S_GE_ for each enhancer was included in the following analysis.

### Construction of minicircle DNA and transfection

The minicircle DNA of eccDNA^chr19:19753606–19753833^ was synthesized by GENERAL BIOL (Anhui, China). The uBMSCs were plated t a density of 2 × 10^5^ cells per well into six-well plates. Cells at a confluence of 60%–80% were transfected with eccDNA using lipo2000 (Biosharp, BL263B) according to the manufacturer’s instructions. After 6 h of transfection, the medium was replaced by iCell mesenchymal stem cell chondrogenic differentiation medium to induce chondrogenic cell differentiation for 4 days and cells were harvested for subsequent experiments. siRNA for PI4KA was designed and synthesized by GenePharma (Shanghai, China). Plasmid for PI4KA was purchased from Wuyuanbio (Tianjin, China). Cells at a confluence of 50%–70% was transfected using lipo2000 (Biosharp, BL263B) according to the manufacturer’s instructions. The cells were collected after 48 h of incubation for DNA or RNA extraction. The sequences used are provided in the Supplementary Material.

### Western blot

Total protein was extracted with radio immunoprecipitation assay (RIPA) lysis buffer (Solarbio, Beijing). The obtained protein concentration was assayed using a bicinchoninic acid assay (BCA) Protein Assay Kit (Thermo). The RIPA lysis buffer containing 1% PMSF was added to the cells. The lysate was transferred to a pre-cooled centrifuge tube and centrifuged at 14,000 rpm for 5 min. The supernatant was transferred to another pre-cooled centrifuge tube and the protein concentration was measured using the BCA protein quantification kit according to the instructions, and 20–40 μg of total protein was taken. The 12% lower layer separation gels and 5% upper layer concentration gels were used according to the manufacturer’s instructions (Wandao, Chengdu). The samples were heated at 95 °C for 5 min in the SDS-PAGE sample loading buffer, separated on the gels, and transferred to the PVDF membranes by a wet transfer instrument (Bio-Rad). The membranes were blocked with 5% BSA for 1 h and then incubated overnight with primary antibodies against PI4KA (polyclonal, IgG) (1:1000, Proteinteck), PI3K (monoclonal, IgG1) (1:1000, Zenbio), p-PI3K (polyclonal, IgG) (1:1000, Affinity), AKT (monoclonal, IgG1) (1:5000, Proteinteck), p-AKT (monoclonal, IgG1) (1:5000, Proteinteck), mTOR (polyclonal, IgG) (1:2000, Proteinteck), p-mTOR (polyclonal, IgG) (1:2000, Proteinteck) and GAPDH (monoclonal, IgG2b) (1:10,000, Proteinteck) were used. After washing, the membranes were incubated with the corresponding secondary antibodies (polyclonal) (1:10,000, Invitrogen) for 2 h.

### Statistics

Two-tailed unpaired t-tests and one-way analysis of variance (ANOVA) were applied using GraphPad Prism 8. R version 4.2.3, Ubuntu 22.04.3 and SRplot [29] were used in data analysis.

## Results

### eccDNA is common in human BMSCs

**Fig. 1:**
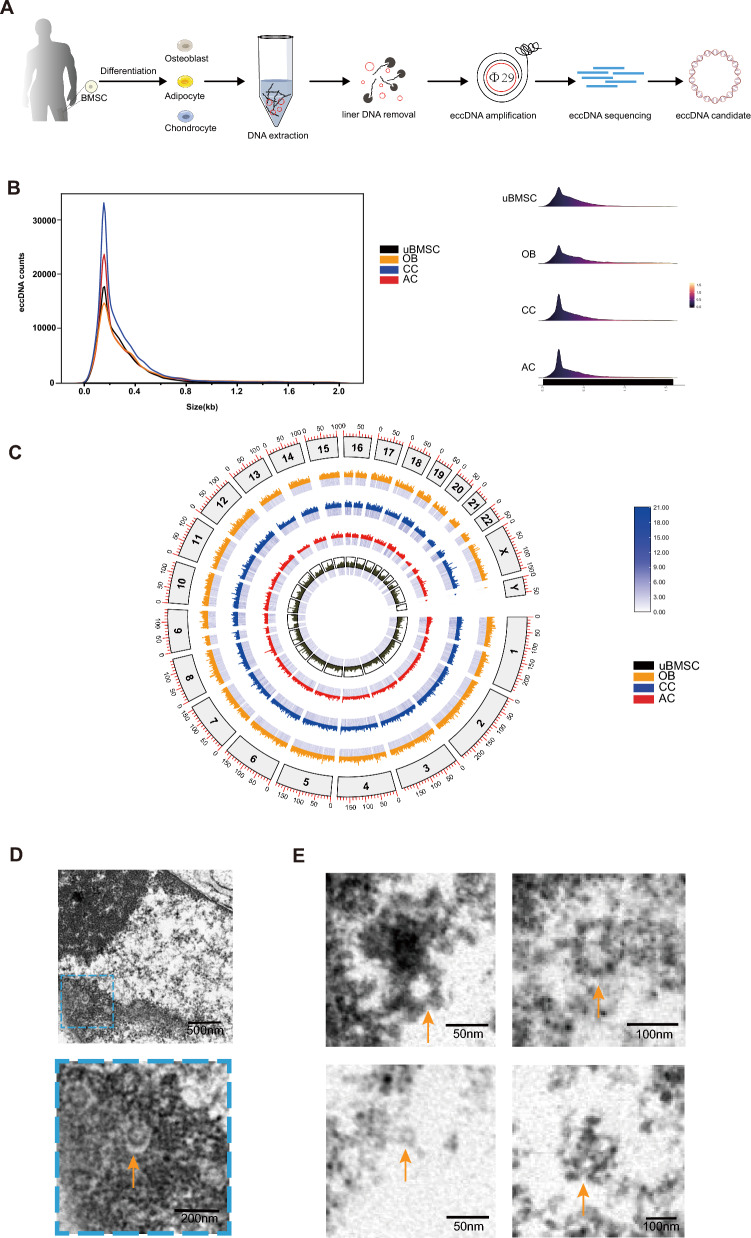
eccDNA is common in human BMSCs. **A,** The Circle-Seq procudure of human BMSCs, OBs, ACs and CCs. **B,** Density plot showing eccDNA size distribution in uBMSCs, OBs, ACs and CCs. **C,** Mapping heatmap showing eccDNA density and distribution on the genome. **D,** TEM of eccDNAs in uBMSCs. **E,** eccDNAs of different sizes in uBMSCs

To gain knowledge about eccDNAs in cell differentiation, we conducted eccDNA sequencing and mapping in human undifferentiated bone marrow mesenchymal stem cells (uBMSCs) and their differentiation into OBs, CCs, and ACs (Fig. S1A-C). eccDNAs were identified using the Circle-seq method [10, 30, 31] (Fig. 1A), which involved DNA extraction, removal of linear DNA, eccDNA amplification using phi29, and subsequent sequencing and mapping of the reads. Overall, we detected 172,966 eccDNAs from 12 samples (51,272 eccDNAs in uBMSC samples, 58,527 eccDNAs in OB samples, 59,306 eccDNAs in AC samples, and 77,906 eccDNAs in CC samples). Notably, a distinct peak of detected eccDNAs was observed near 188 bp (Fig. 1B), and the three differentiated groups exhibited a higher number of eccDNAs compared to uBMSCs (Fig. S2). The normalized genomic distribution of eccDNAs showed variation in density across different chromosomes. Gene-rich chromosomes like chromosome 19 generate more eccDNAs [10, 32]. The varying eccDNA quantities highlight their heterogeneity across different chromosomes and groups, which is critical for understanding the biological generation of roles of eccDNA. The chromosome 19 generates more eccDNAs, which is consistent with previous studies and potentially linked to its unique genomic features like high gene density or repetitive sequences [10, 33]. The genome mapping revealed that eccDNAs were widely distributed throughout the entire genome (Fig. 1C).

To visualize the definitive structures of eccDNA in normal cells, we captured images of eccDNAs in human BMSCs using TEM. The TEM images revealed circular extrachromosomal DNA molecules, confirming the circular nature of eccDNAs in BMSCs and supporting their existence in normal cell lines (Fig. 1D, E).

### eccDNA increases gene transcription

**Fig. 2:**
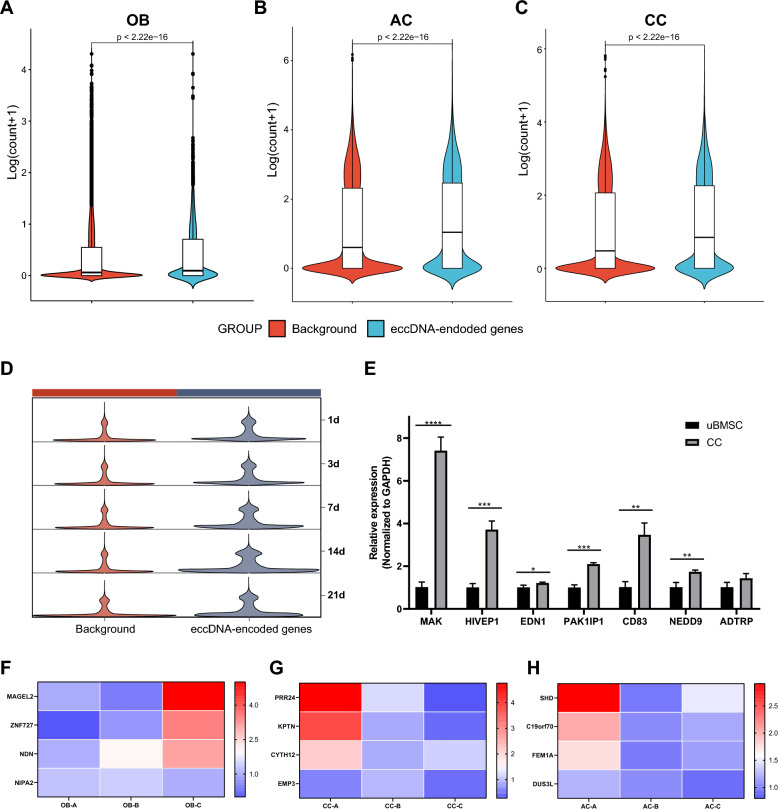
eccDNA increases transcription levels. **A-C,** Violin plots showing the increased expression of eccDNA-encoded genes in OB, AC and CC groups. **D,** Comparison of the expression of the eccDNA-encoded genes with the background in different stages of chondrogenic differentiation. **E,** The expression of eccDNA-encoded genes on eccDNAchr6:10523673–18078112 in CC and undifferentiated groups, n = 3, *p < 0.05; **p < 0.01, ***p < 0.001, ****p < 0.0001. **F–H,** Comparison of the expression of eccDNA-encoded genes in samples with different amount of eccDNAs. For corresponding eccDNA-encoded genes, samples with more eccDNAs exhibited higher gene expression compared to other samples with less or no eccDNAs. The color scale represents the relative expression of the genes which is normalized to GAPDH

We then identified lineage-specific eccDNAs and obtained 31,330 OB-specific eccDNAs, 21,923 AC-specific eccDNAs and 36,264 CC-specific eccDNAs (Fig. S3). To investigate the transcription of genes on eccDNA in normal cells, we conducted RNA-seq for the samples in uBMSCs and OBs. We proposed that, for entire genes on eccDNAs, referred as eccDNA-encoded genes, eccDNA promotes their expression, like ecDNA-oncogene in cancer cells. To validate this, we first compared the expression of the eccDNA-encoded genes with the background (all genes). The results showed the expression of eccDNA-encoded genes was higher than the background in OB, AC and CC groups (Fig. 2A-C). To enhance the universality, we adopted external datasets including RNA-seq data of ACs and CCs [22–24]. The results from public datasets showed the expression of eccDNA-encoded genes was also higher than the background (Fig. S4). To examine the time difference between the eccDNA and RNA levels, we next explored the transcriptional profiles of eccDNA-encoded genes in different stages of chondrogenesis. The expression of the eccDNA-encoded genes was compared with the background at 1, 3, 7, 14 and 21 days of chondrogenesis. Although all investigated stages showed differences between the eccDNA encoded genes and the background, the most significant differences appeared at 14 days, the time of cell differentiation for Circle-seq, suggesting temporal concordance between the eccDNA and RNA levels (Fig. 2D). Next, the expression of several eccDNA-encoded genes was validated by RT-qPCR. eccDNA^chr6:10523673–18078112^ was found in all CC samples and absent in uBMSC samples. We compared the expression of several genes on eccDNA^chr6:10523673–18078112^ in CC group and undifferentiated group. We explored the expression of 7 entire genes, including male germ cell associated kinase (MAK), human immunodeficiency virus type I enhancer binding protein 1 (HIVEP1), endothelin 1 (EDN1), P21-activated protein kinase-interacting protein 1 (PAK1IP1), cluster of differentiation 83 (CD83), neural precursor cell expressed developmentally down-regulated protein 9 (NEDD9) and androgen dependent TFPI regulating protein (ADTRP), on eccDNA^chr6:10523673–18078112^ in CC and uBMSC group. We found most of them were upregulated in CC group compared to uBMSC group (Fig. 2E). To exclude the effects of differentiation process on gene expression, we investigated the intra-group expression of eccDNA-encoded genes. The expression of genes in samples with more corresponding eccDNAs and samples with less corresponding eccDNAs were compared in the same groups. For 3 samples (noted as A, B and C) in each group, some eccDNAs with entire genes were more abundant in one sample than the other two samples, such as melanoma antigen family member L2 (MAGEL2), zinc finger protein 727 (ZNF727), necdin (NDN) and non-imprinted in prader-willi/angelman syndrome region protein 2 (NIPA2) in OB-C sample, proline rich 24 (PRR24), kaptin (KPTN), cytohesin 2 (CYTH2) and epithelial membrane protein 3 (EMP3) in CC-A sample, Src homology 2 domain containing transforming protein D (SHD), chromosome 19 open reading frame 70 (C19orf70), fem-1 homolog A (FEM1A) and dihydrouridine synthase 3 like (DUS3L) in AC-A sample, and we explored the expression of these genes. The results showed that more eccDNAs induce higher gene expression (Fig. 2F-H).

### eccDNA in human BMSCs contains highly accessible chromatin

**Fig. 3:**
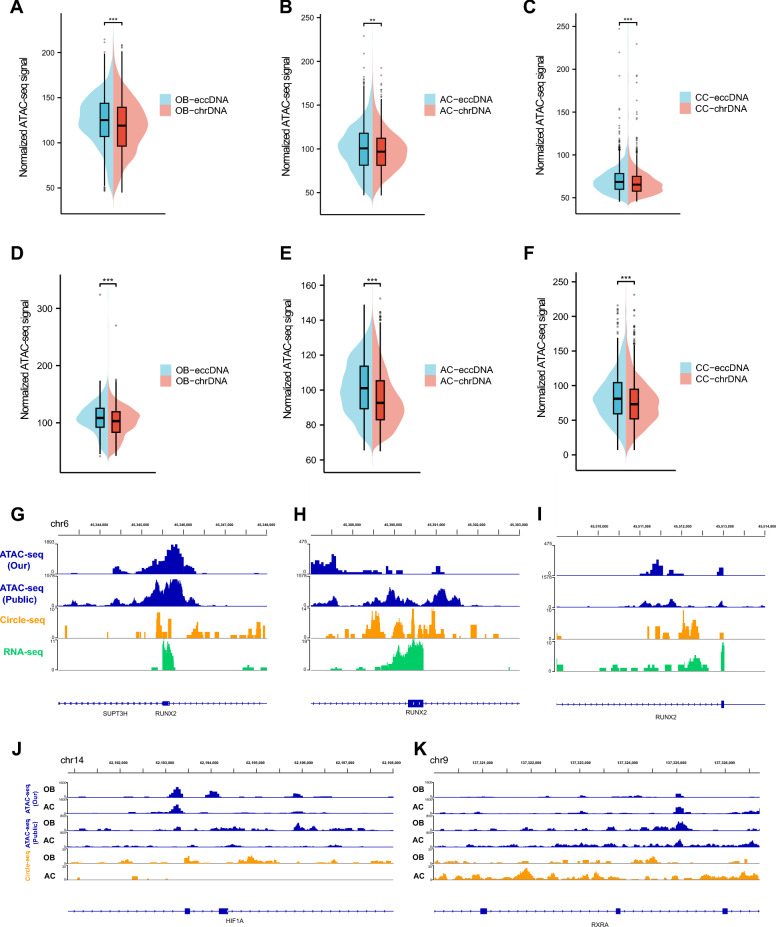
The chromatin landscape of eccDNA. **A-C,** Comparison of the ATAC-seq signal between eccDNA and chrDNA in OB, AC and CC groups using our ATAC-seq data. **D-F,** Comparison of the ATAC-seq signal between eccDNA and chrDNA in OB, AC and CC groups using public ATAC-seq data. **G-I,** Genome browser screenshots showing the ATAC-seq, eccDNA and transcription profiles of different exons of RUNX2. **J,K,** Genome browser screenshots presenting the differential ATAC-seq and eccDNA profiles of OB functional gene HIF1A and AC functional gene RXRA in OB and AC groups

Most of the chromosomal DNA (chrDNA) is tightly packed and inaccessible to transcription [34]. It has been reported that eccDNA in cancer cells with less compacted organization is more accessible than chrDNA [6]. To confirm whether eccDNA in human BMSCs presents similar structure, we explored their chromatin landscape. We used the ATAC-seq to assess chromatin accessibility and to map nucleosome positions [3, 25, 26]. The analysis using our ATAC-seq and public ATAC-seq data showed consistent results that eccDNA displayed a significantly higher ATAC-seq signal (Fig. 3A-F), which suggests that the eccDNA chromatin landscape is more accessible than chrDNA in human BMSCs. We further validated it on differentiation functional genes. RUNX2 is a key transcription factor which is essential for osteogenic differentiation of BMSCs [1]. By integrating ATAC-seq profiles with eccDNA and RNA-seq data, we found the high ATAC-seq signals in the eccDNA contribute to the transcription of RUNX2 (Fig. 3G-I).

The lineage determination between osteogenic and adipogenic differentiation of MSCs is under the control of the delicate balance among different transcription factors [35, 36]. We next investigated two MSC fate regulators, including osteoblasts functional gene hypoxia inducible factor 1 subunit alpha (HIF1A) and adipocytes functional gene retinoid X receptor alpha (RXRA) [3, 36]. The results showed more eccDNAs formed in the corresponding functional genes in OBs or ACs, with higher accessible chromatin compared to the other (Fig. 3J, K). These results highlighted the relationship of increased transcription and high accessibility of eccDNA, bridging eccDNA features with biological function.

### PI4KA promotes the osteogenesis of BMSCs

**Fig. 4:**
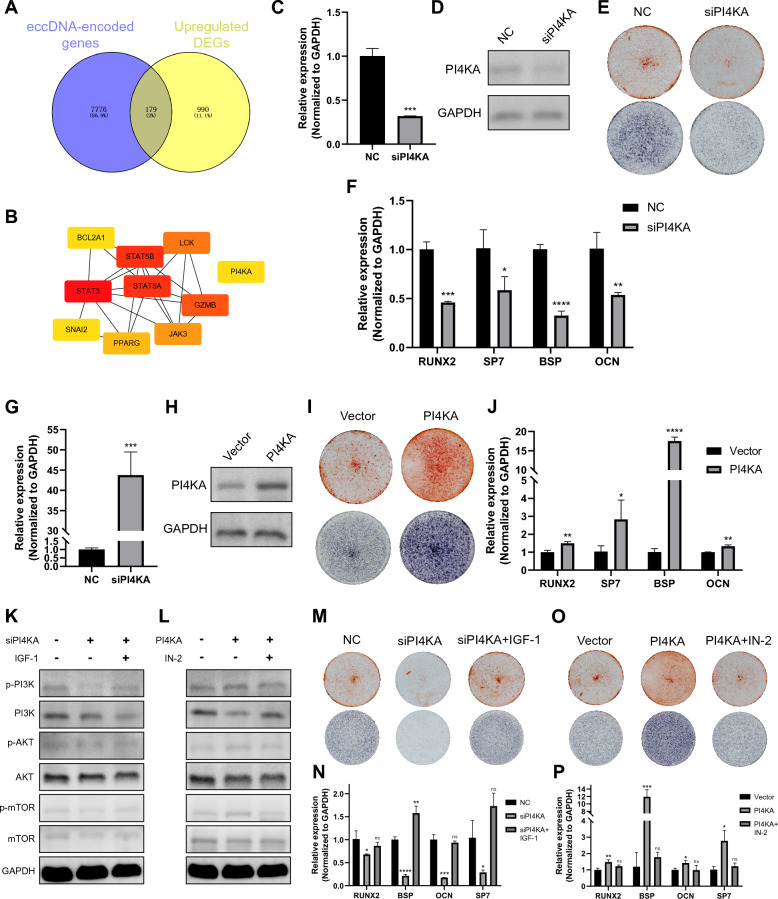
PI4KA promotes the osteogenic differentiation of human BMSCs via the PI3K/AKT/mTOR pathway. **A,** Venn plot identifying the upregulated eccDNA-encoded genes in OBs. **B,** The Top 10 hub genes by cytoscape. **C, D,** Detection of PI4KA mRNA and protein expression levels after transfection of siRNA, n = 3, ***p < 0.001. **E,** ARS and ALP staining of PI4KA knockdown human BMSCs and control group. **F,** The mRNA expression of osteogenic markers RUNX2, SP, BSP and OCN in PI4KA knockdown and control groups, n = 3, *p < 0.05; **p < 0.01, ***p < 0.001, ****p < 0.0001. **G,H,** Detection of PI4KA mRNA and protein expression levels after transfection of plasmid. **I,** ARS and ALP staining of PI4KA overexpression human BMSCs and control group. **J,** The mRNA expression of osteogenic markers RUNX2, SP, BSP and OCN in PI4KA overexpression and control groups, *p < 0.05; **p < 0.01, ***p < 0.001, ****p < 0.0001. **K,** The levels of proteins in PI3K/AKT/mTOR pathways after PI4KA knockdown and reversed by IGF-1. **L,** The levels of proteins in PI3K/AKT/mTOR pathways after PI4KA overexpression and reversed by IN-2. **M,** ARS and ALP staining after PI4KA knockdown and reversed by IGF-1. **N,** The mRNA expression of osteogenic markers RUNX2, SP, BSP and OCN after PI4KA knockdown and reversed by IGF-1, *p < 0.05; **p < 0.01, ***p < 0.001, ****p < 0.0001. **O,** ARS and ALP staining after PI4KA overexpression and reversed by IN-2. **P,** The mRNA expression of osteogenic markers RUNX2, SP, BSP and OCN after PI4KA overexpression and reversed by IN-2, *p < 0.05; **p < 0.01, ***p < 0.001, ****p < 0.0001. The full-length blots (D, H, K, L) were presented in Supplementary Fig. 4

To further elucidate the roles of eccDNA in MSC differentiation, we investigated eccDNA-encoded genes in osteogenesis by Circle-seq and RNA-seq data. Totally, we obtained 179 up-regulated eccDNA-encoded genes by intersecting eccDNA-encoded genes and up-regulated differentially expressed genes (DEGs) (Fig. 4A). We constructed the protein–protein interaction network for the 179 genes by STRING database and 10 hub genes were identified using cytoscape (Fig. 4B) [37, 38]. The 10 hub genes clustered into two groups, one containing 9 genes, most of which are known to regulate osteogenesis, like STAT3, STAT5A and PPARG [2, 39, 40]. However, the role of PI4KA, an endoplasmic reticulum-resident enzyme to convert phosphatidylinositol to PI4P, in osteogenesis is unrevealed [41]. We next determined the roles of PI4KA in osteogenic differentiation. PI4KA expression was inhibited by siRNA (Fig. 4C, D). Knockdown of PI4KA weakened the intensities of ALP and Alizarin Red staining (ARS) in human BMSCs (Fig. 4E). The expression of osteogenic markers RUNX2, SP7, BSP and OCN was also suppressed in PI4KA knockdown BMSCs compared to the control group (Fig. 4F). The overexpression of PI4KA yielded the opposite effects. PI4KA overexpression induced enhanced intensities of ARS and ALP, and increased expression of the osteogenic markers (Fig. 4G-J). These results demonstrated PI4KA is a positive regulator in the osteogenic differentiation of human BMSCs.

PI4KA contributes to the synthesis of PI(4,5)P2, which is a precursor of the PI3K lipid substrate [41, 42]. By rescue experiments, we found PI4KA regulate osteogenesis via the PI3K/AKT signaling pathway (Fig. 4K-P). PI4KA knockdown suppressed the PI3K/AKT/mTOR signaling and osteogenic differentiation of human BMSCs, which was reversed by IGF-1, a PI3K/AKT activator [43]. On the contrary, overexpression of PI4KA activated the PI3K/AKT/mTOR pathway and osteogenic differentiation of human BMSCs, which was reversed by the inhibitor PI3K/AKT/mTOR-IN-2 (IN-2) [44].

### eccDNA functions as enhancer to promote the differentiation of BMSCs

**Fig. 5:**
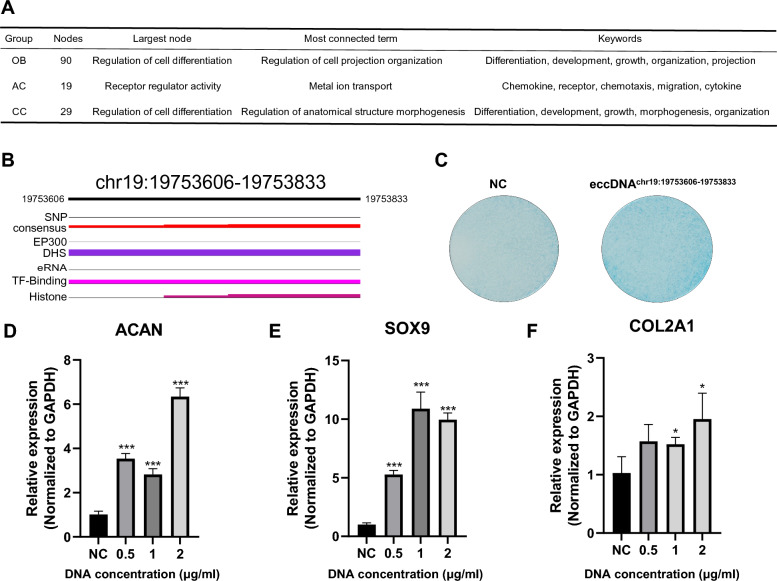
eccDNAs function as enhancers to promote human BMSC differentiation. **A**, Information of the largest biological clusters of eccDNA-regulated genes in OB, AC and CC groups. **B,** Enhancer related signals on eccDNA^chr19:19753606–19753833^. **C,** Alcian blue staining after 4 days of transfection of eccDNAchr19: 19753606–19753833. **D-F,** Chondrogenic markers were upregulated after transfection of eccDNAchr19: 19753606–19753833 in a dose-dependent manner, n = 3, *p < 0.05; **p < 0.01, ***p < 0.001

It has been reported that eccDNA also functions as mobile enhancers in cancer genome [11, 12, 45]. We next identified eccDNAs containing entire enhancers and corresponding gene targets, noted as eccDNA-regulated genes, by GeneHancer and EnhancerAtlas [27, 28]. Pathway analysis of these eccDNA-regulated genes was conducted in the three differentiated groups. To reduce redundancy of the enriched terms, we used vissE to identify specific biological directionality of these genes [46]. The largest biological clusters of eccDNA-regulated genes in OB, AC and CC were all differentiation-related (Fig. 5A). To validate the role of the eccDNA as enhancer in cell differentiation, we synthesized eccDNA^chr19:19753606–19753833^ in vitro by minicircle DNA method [47]. eccDNA^chr19:19753606–19753833^ contains enhancer sequences (Fig. 5B) and was found in all samples in the CC group. Transfection of eccDNA^chr19:19753606–19753833^ promoted chondrogenic differentiation of hBMSCs and marker genes of chondrogenic differentiation were upregulated in dose-dependent manner (Fig. 5C-F). These results provide evidence that eccDNAs can function as enhancers to promote the differentiation of BMSCs.

## Discussion

eccDNAs are prevalent in the cancer genome [5–7]. Our study confirms the existence of eccDNA in hBMSCs, indicating that eccDNA not only plays an important role in tumor cells but also in normal cells. The circular structure of eccDNA may confer higher stability, allowing it to persist in cells for a long time and participate in gene regulation [6]. Circle-seq analysis revealed the dynamic changes of eccDNA during the differentiation process of hBMSCs. The number of eccDNA in the differentiation groups (OBs, ACs, and CCs) was higher than that in the undifferentiated group, which might be due to the increased genomic activity and instability during cell differentiation. This is related to active DNA replication, transcription, and chromatin remodeling during differentiation, which may lead to more DNA breaks and circularization events [48, 49]. Additionally, the distribution characteristics of eccDNA suggest that eccDNA may be closely related to local genomic features. Particularly, more eccDNA was produced in gene-enriched regions such as chromosome 19, suggesting that the formation of eccDNA may be significantly influenced by gene-related activities.

Oncogenes on eccDNAs achieve high copy numbers and the highly accessible chromatin on eccDNA further facilitates the oncogene expression [6]. Our study demonstrated similar mechanisms underlying the differentiation of human BMSCs. In differentiated groups including OBs, ACs and CCs, eccDNA-encoded genes presented higher expression compared to the background. Although the chromosomal gene activity could contribute to the gene expression, the chromosomal gene expression likely has minimal impact on the results as the chromosomal gene expression is nearly the same level for the compared genes if the comparison was in the same group [6]. With the development of related knowledge and technology, future study could silence the chromosomal copies of the genes solely to provide an eccDNA-specific perspective. The ATAC-seq data showed overall higher signal on eccDNA than chromosomal DNA. Differentiation functional genes were found amplified on eccDNA with enhanced chromatin accessibility and highly transcribed, such as RUNX2, a key transcription factor for BMSC osteogenesis. Moreover, eccDNAs with enhancers are responsible for BMSC differentiation by regulating differentiation-related genes. These results revealed the mechanism by which eccDNA contributes to BMSC differentiation. We speculate that similar mechanisms exist in other biological processes. It should be noted that the ATAC-seq does not distinguish signals between chromosomal DNA and eccDNA, and its short read lengths (50–100 bp) limit the detection of eccDNA-specific circular features such as circular junction points. Future studies could integrate long-read sequencing for a more comprehensive characterization of the open chromatin states on eccDNA.

The non-negligible roles of eccDNAs in BMSC differentiation indicate novel regulators for stem cell differentiation from the view of eccDNA. We combined Circle-seq and RNA-seq data and identified a potential candidate for osteogenic differentiation of human BMSCs, PI4KA. PI4KA is an essential lipid kinase and regulates the phosphatidylinositol 4-phosphate and phosphoinositide signaling at the plasma membrane [41, 42]. However, the role of PI4KA in osteogenic differentiation is unrevealed. We found PI4KA regulates the osteogenesis of human BMSCs via the PI3K/AKT/mTOR pathway. This finding directly links phospholipid metabolism process with cell fate determination, providing new insights into the coordinated regulation of metabolism and signal transduction in cell differentiation. We found that PI4KA activates the PI3K/AKT/mTOR pathway in osteogenesis, which not only confirmed the pivotal role of this classic signaling pathway in osteogenic differentiation but also revealed the function of PI4KA on the signal transduction network. The results provide a potential target for optimizing osteogenic differentiation strategies of hBMSCs. Currently, clinical applications of bone tissue engineering often face bottlenecks such as low osteogenic efficiency and high heterogeneity [50, 51]. By regulating PI4KA activity or interfering with its downstream effector molecules, new induction differentiation schemes may be established. Notably, abnormal PI3K/AKT signaling is common in metabolic bone diseases such as osteoporosis. This study suggests that PI4KA dysfunction may be a potential pathological mechanism, opening up new ideas for the development of small molecule drugs targeting phospholipid metabolism. The regulatory axis discovered in this study also provides insights into biomarker development. In addition, the lipid kinase-classic pathway interaction pattern revealed in this experiment can provide a reference model for researches on differentiation regulation of other stem cells. Subsequent studies are needed to analyze the dynamic distribution characteristics of PI4KA-mediated phospholipid metabolites in cells at the spatial level and verify their repair efficacy in bone defect models. These results provide novel insights into BMSC differentiation and signature exploitation.

Enhancers are key elements in the regulation of gene transcription, activating or inhibiting gene expression by interacting with promoters. Enhancers play a key role in cell differentiation by regulating the expression of differentiation-related genes [52]. In recent years, studies have found that eccDNA can carry functional enhancer sequences and act as mobile enhancers to regulate the expression of oncogenes in cancer [7, 11, 53]. Enhancers on eccDNA can interact with promoters in the host genome through chromatin looping, thereby driving the high expression of oncogenes [7, 45]. However, it remains unclear whether eccDNA can function as enhancers in cell differentiation and its impact on gene regulation. This study found that eccDNA-regulated genes were significantly enriched in differentiation-related biological functions in OBs, ACs, and CCs. These results suggest that eccDNA may play an important role in cell differentiation by carrying functional enhancer sequences and regulating the expression of differentiation-related genes. We also found that eccDNA^chr19:19753606–19753833^ could promote the chondrogenic differentiation of hBMSCs, and the expression of chondrogenic differentiation markers is dose-dependently upregulated. The results indicate that eccDNA regulates the expression of chondrogenic differentiation-related genes by carrying functional enhancer sequences, thereby promoting the chondrogenic differentiation of hBMSCs. This provides an important theoretical basis for understanding the function of eccDNA as enhancers in cell differentiation and its potential applications in tissue engineering and regenerative medicine. However, we have not found specific eccDNAs functioning as enhancers to regulate the osteogenesis and adipogenesis of BMSCs by experimental validation, which remains further exploration in the future study. Overall, our study provides new insights into the role of eccDNA in BMSC differentiation.

## Conclusions

In this study we demonstrate that eccDNA is common in human BMSCs. eccDNA regulates BMSC differentiation by increasing gene copy numbers, chromatin accessibility and functioning as enhancers. Our study provides the evidence of eccDNA in regulating MSC differentiation, which will be helpful for further research and clinical applications.

## Supplementary Information

Additional file 1.

Additional file 2.

Additional file 3.

Additional file 4.

Additional file 5.

Additional file 6.

Additional file 7.

Additional file 8.

## Data Availability

The Circle-seq data was deposited in the Gene Expression Omnibus with number GSE261856. Other data that support the findings of this study are available from the corresponding author upon reasonable request.
