# Supplementary figures and images for: Extrachromosomal circular DNAs in the differentiation of human bone marrow mesenchymal stem cells
Source: Stem Cell Res Ther. 2025 Jul 18;16:383. doi: 10.1186/s13287-025-04516-x (PMC12275313; doi:10.1186/s13287-025-04516-x)

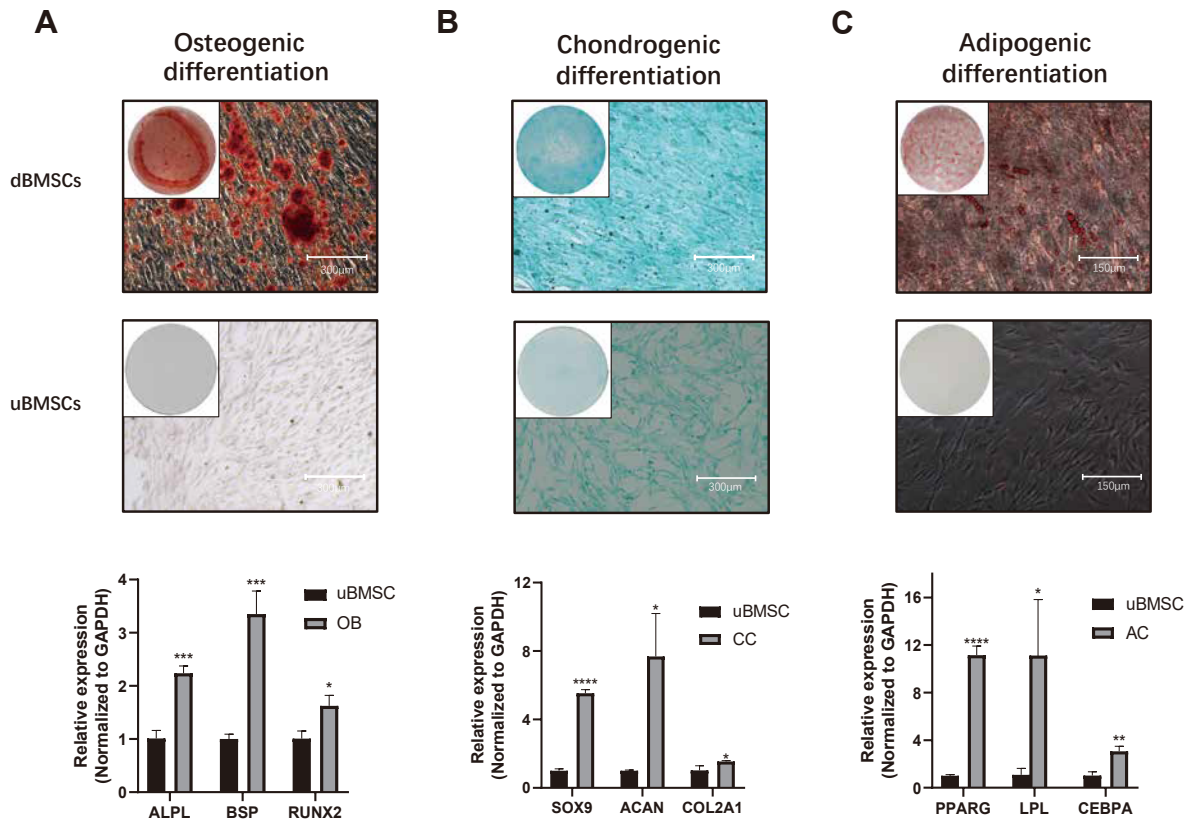

**Figure S1**

Supplement: Supplementary file 1 — Additional file 1. [file 13287_2025_4516_MOESM1_ESM.pdf]

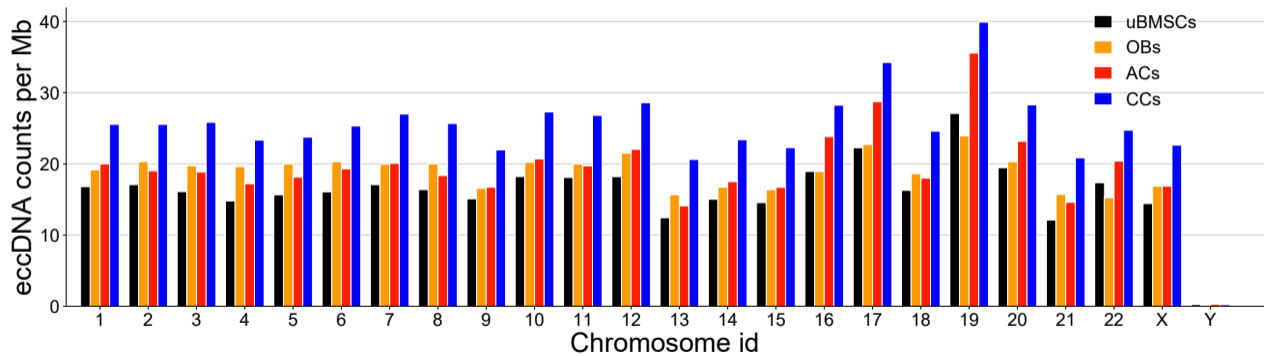

**Figure S2**

Supplement: Supplementary file 2 — Additional file 2. [file 13287_2025_4516_MOESM2_ESM.pdf]

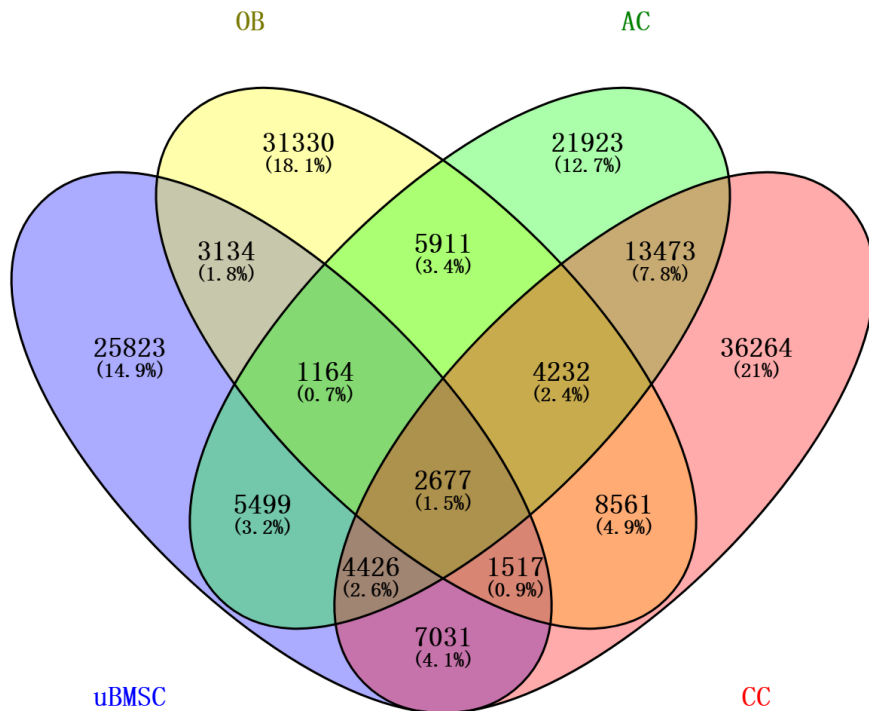

**Figure S3**

Supplement: Supplementary file 3 — Additional file 3. [file 13287_2025_4516_MOESM3_ESM.pdf]

**A**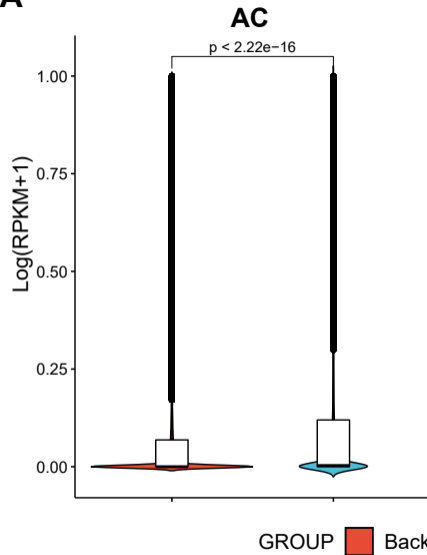**B**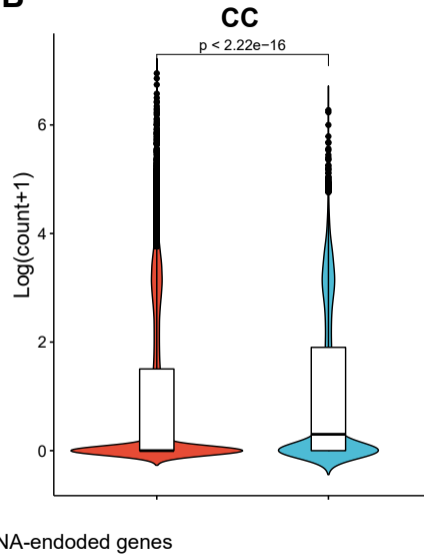**Figure S4**

Supplement: Supplementary file 4 — Additional file 4. [file 13287_2025_4516_MOESM4_ESM.pdf]

Figure 4D

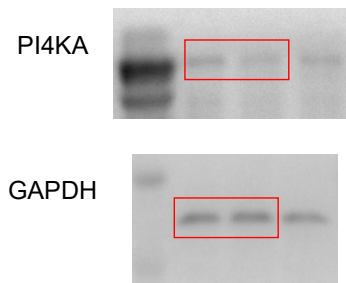

Figure 4H

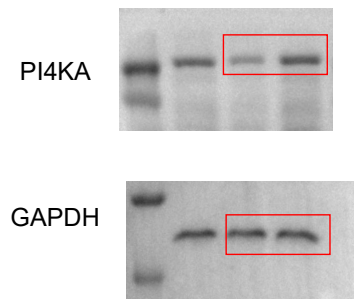

Figure 4K

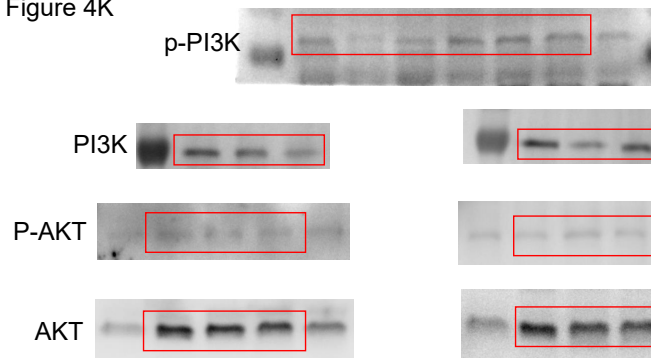

Figure 4L

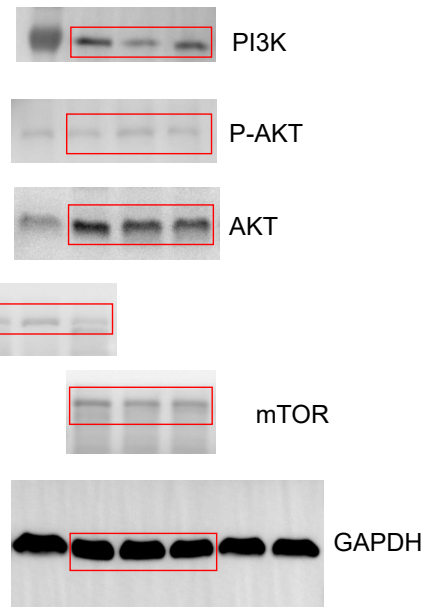

GAPDH

Figure S5

Supplement: Supplementary file 5 — Additional file 5. [file 13287_2025_4516_MOESM5_ESM.pdf]
