# Supplementary material for: Extrachromosomal circular DNAs in the differentiation of human bone marrow mesenchymal stem cells
Source: Stem Cell Res Ther. 2025 Jul 18;16:383. doi: 10.1186/s13287-025-04516-x (PMC12275313; doi:10.1186/s13287-025-04516-x)
Supplement: Supplementary file 6 — Additional file 6. [file 13287_2025_4516_MOESM6_ESM.pdf]

Supplementary Materials for

Extrachromosomal circular DNAs in the differentiation of human bone marrow mesenchymal stem cells

Yuxi Gu, Yidan Song, Shuhua Wang, Jun Liu*
Corresponding author: Jun Liu,
junliu@scu.edu.cn

Figure S1. Osteogenesis, chondrogenesis and adipogenesis abilities of human BMSCs. A, Alizarin Red S staining and RT-qPCR analysis of osteogenic genes (ALPL, BSP and RUNX2) after 14-day osteogenic induction, n=3, *p< 0.05; **p < 0.01, ***p < 0.001. **B**, Alcian blue staining and qRT-PCR analysis of chondrogenic genes (SOX9, ACAN and COL2A1) after 14-day chondrogenic induction, n=3, *p< 0.05; **p < 0.01, ***p < 0.001, ****p < 0.0001. **C**, Oil red o staining and qRT-PCR analysis of adipogenic genes (PPARG, LPL and CEBPA) after 14-day adipogenic induction, n=3, *p< 0.05; **p < 0.01, ***p < 0.001, ****p < 0.0001.

Figure S2. eccDNA frequency relative to chromosome. eccDNA/Mb per chromosome from uBMSCs, OBs, ACs and CCs.

Figure S3. Identification of lineage-specific eccDNAs.

Figure S4. Violin plots showing the increased expression of eccDNA-encoded genes in AC (A) and CC (B) groups

Figure S5. Original WB.

Table S1. List of siRNA sequences.

Table S2. List of RT-qPCR primer sequence.
